# Glass ionomer fissure sealants versus fluoride varnish application on children’s behaviour: a randomised controlled trial

**DOI:** 10.1007/s40368-024-00952-0

**Published:** 2024-11-04

**Authors:** H. H. C. Chiu, P. P. Y. Lam, C. K. Y. Yiu

**Affiliations:** Paediatric Dentistry, Faculty of Dentistry, The University of Hong Kong, 2nd Floor, Prince Philip Dental Hospital, 34 Hospital Road, Sai Ying Pun, Pokfulam, Hong Kong SAR

**Keywords:** Pit and fissure sealants, Fluoride varnish, Dental anxiety, Pre-school children, Randomised controlled trial

## Abstract

**Purpose:**

Glass ionomer fissure sealant (GIS) and fluoride varnish (NaFV) are two preventive interventions applicable to pre-school children. However, their application effects on young children’s behaviour are understudied. The study compared the effects of GIS and NaFV applications on dental anxiety (DA), cooperativeness and pain level in pre-school children when applied to primary second molars in outreach settings.

**Methods:**

Four hundred and thirteen children were recruited for the study, out of which 228 were allocated to NaFV group whilstand 185 allocated to the GIS group. One calibrated examiner screened and randomly allocated the children into either group. Another calibrated examiner recorded the children’s DA level and cooperativeness with Frankl Behaviour Rating Scale (FBRS) and Venham Behaviour Rating Scale (VBRS) throughout the treatment period. Subjects self-reported their pain levels using Wong–Baker Faces Scale (WBFS).

**Results:**

Significant difference in postoperative anxiety was found using VBRS, with children in NaFV group having more positive scores post-treatment (*p* = 0.016). Only the type of preventive interventions significantly affected postoperative DA and patient cooperation (*p* = 0.032), whereas no other clinical findings and socio-demographic factors significantly influenced the children’s behaviour postoperatively.

**Conclusion:**

NaFV application is associated with less DA and more cooperative behaviour in pre-school children compared to GIS application although similar pain levels were recorded.

## Introduction

Dental caries is a multifactorial and dynamic disease that results in demineralization of dental hard tissues (Tinanoff et al. 2019). The prevalence of early childhood caries (ECC), especially in young children, is high, ranging from 12 to 98% (Tinanoff et al. 2019) in 4-year-old children in different countries. Considering the high prevalence of active and untreated decay in young children (Tinanoff et al. 2019), the importance of secondary prevention must not be underestimated. Secondary prevention comprises preventing progression of the disease and stimulating remineralization of initial lesions (Tinanoff et al. 2019). Fundamental cornerstones to secondary prevention include professional topical fluoride application and sealing occlusal surfaces of molars to arrest caries progression (Ahovuo-Saloranta et al. 2016; Marinho et al. 2013).

Fissure sealants could penetrate susceptible surfaces of teeth to physically prevent food trapping and biofilm stagnation which may induce caries progression. Furthermore, the current evidence has illustrated the effectiveness of sealants to minimise progression of non-cavitated occlusal caries lesions (Ahovuo-Saloranta et al. 2017). On the other hand, current literature also recognises the effectiveness of fluoride varnish to prevent dental caries progression, with a Cochrane systematic review reporting a prevented fraction at 43% in the permanent dentition (Marinho et al. 2013). Another systematic review also identified 5% sodium fluoride varnish was the most efficacious for arresting or reversing non-cavitated smooth surfaces lesions (Urquhart et al. 2019) amongst other non-restorative treatment for dental caries. Considering the efficacy of both modalities, existing research has found no significant differences in effectiveness between fluoride varnish and fissure sealant in preventing the progression of dental caries in both primary and permanent dentitions (Kashbour et al. 2020; Lam et al. 2020).

When providing the above-mentioned treatments to the paediatric population, clinicians must also consider the effect dental anxiety (DA) and fear. Dental fear is a normal reaction to an intimidating stimulus in a dental setting (Klingberg and Broberg 2007) whilst DA is a state of apprehension towards something threatening that is going to happen related to dental treatment (Klingberg and Broberg 2007). It is common in different populations, with reported prevalence ranging from 5.7 to 19.5% in children and adolescents (Klingberg and Broberg 2007). Existing literature proposed some factors which increases the likelihood of DA and dental behaviour management problems (DBMP), such as general fear (Klingberg et al. 1995), temperament (Arnrup et al. 2002) and painful experiences from treatment (Raadal et al. 2002). Dental anxiety and DBMP may pose a problem for paediatric dentists and could be a barrier to carrying out successful treatment (Ashley et al. 2018). Hence, considering the conjoining impact of DA and ECC, it is important for paediatric dentists to investigate which treatment modalities evoke less anxiety, less pain and promote cooperativeness so that treatment could be delivered smoothly and safely. Furthermore, clinicians may gain insights to providing a favourable experience and building a positive attitude towards dental treatment for the patient in the long run.

A recent randomised controlled trial reported that quarterly application of fluoride varnish and a single application of glass ionomer sealants demonstrated comparable effectiveness in preventing occlusal caries in primary molars (Lam et al. 2021). However, there are currently limited studies that compared the effect of different preventative modalities for caries management on children’s behaviour. Hence, the present study aims to compare DA, pain associated and application time of fissure sealant application versus topical fluoride varnish application in pre-school children. The null hypotheses being tested were:

1. No difference in change of DA levels between placement of glass ionomer fissure sealant (GIS) and topical application of 5% sodium fluoride varnish (NaFV) in pre-school children.
2. No difference in change of pain levels between placement of GIS and NaFV application in pre-school children.
3. No difference in application time of NaFV and GIS on second primary molars.

## Material and methods

The parallel group randomised controlled trial was conducted in Hong Kong, China. Ethical approval was obtained from the Institutional Review Board (IRB) of the University of Hong Kong/Hospital Authority Hong Kong West Cluster (UW 18–053). The trial was registered in ClinicalTrials.gov (NCT04163354).

### Sample size calculation

The sample size was calculated based on the outcome of child anxiety, cooperativeness and pain assessed using Frankl Behaviour Rating Scale (Frankl 1962) (FBRS), Venham Behaviour Rating Scale (Venham et al. 1980) (VBRS) and Wong–Baker Faces Scale (Wong and Baker 2001) (WBFS). Based on Yon et al. (2020a, b), the proportion of pre-school children having negative dental behaviours is 4.4%. Assuming that quarterly application of NaFV can completely alleviate DA of pre-school children whilst the GIS group could not achieve the same outcome, the sample size was 180 per group when targeting a significance level of 0.05 and statistical power of 0.9; i.e. a total of 360 children at baseline. The initial sample size is further enlarged by 10% as children was further excluded if GIS cannot be placed on their occlusal surfaces. Hence, a total of 396 children were required, with 195 children being allocated to each group.

The children underwent additional follow-up to evaluate the development of occlusal caries in their primary molars over a period of 18–24 months. When the sample size was calculated based on progression of occlusal dentinal caries, it was determined that 284 children were required at baseline. The methodology for calculating the sample size is detailed elsewhere (Lam 2023). Considering both calculations, a total of at least 396 children were needed for this study.

### Subject selection

Children aged between 3 and 6 years attending kindergarten grade 1 and nursery grade 2 in Hong Kong were invited to participate in the clinical trial. The study was conducted in 18 preschools located in various districts in Hong Kong using a combination of stratified and purposive sampling methods [Hong Kong Island (*n* = 4), Kowloon (*n* = 5), New Territories West (*n* = 5), New Territories East (*n* = 4)]. Parents or legal guardians received an invitation letter explaining the objectives and the procedures of the study. Only those children whose parents or legal guardians had signed the informed consent were included. In addition, parents were asked to complete a questionnaire regarding their socio-economic background and oral health-related behaviours.

### Inclusion criteria

The potential subjects were screened using the following methods: plaque assessment using Visible Plaque index (VPI) (Ainamo and Bay 1975), overall tooth status assessment with diagnostic criteria recommended by WHO 2013(WHO 2013) and dental caries status cased on ICDAS code score (Ismail et al. 2007). After screening, children considered to be moderate to high caries risk according to the criteria (“Guideline on Caries-risk Assessment and Management for Infants, Children, and Adolescents,” 2016) outlined by the American Academy of Paediatric Dentistry (AAPD) were included. The criteria were children who were recent immigrants or of lower socio-economic background, who snacked more than 3 times a day, children with visible plaque, or who were put to sleep with a bottle containing natural or added sugar and children having decayed-missing-filled surfaces score of more than 1, having visible plaque or enamel defects. According to the AAPD guidelines, children with either moderate or high caries risk are recommended to receive fissure sealants and topical fluoride varnish application regularly every 3 months (“Guideline on Caries-risk Assessment and Management for Infants, Children, and Adolescents,” 2016).

### Exclusion criteria

Children with serious systemic diseases requiring long-term medication or special needs were excluded. Furthermore, children who were uncooperative during the procedure or had received professional topical fluoride treatment in the past 6 months were excluded. Furthermore, primary second molars that are partially erupted, with enamel defects or with sealants, restorations or dentinal caries indicated by ICDAS scores 4, 5, and 6 was excluded.

### Subject recruitment

Subject recruitment lasted from September to December 2019. Children attending kindergarten grade 1–2 in 16 study kindergartens were approached. A total of 486 parental consents and completed questionnaires were obtained. Out of the 486 children, only 413 were deemed eligible and randomly assigned into the two treatment groups.

### Questionnaire

Parents were required to complete a validated questionnaire before the baseline examination and intervention. The questionnaire included the child’s personal information, such as gender, age, place of birth and medical history; oral health-related behaviours, such as snacking habit, frequency and parental supervision of toothbrushing, use of toothpaste and night-time bottle habit; the child’s socio-economic background, such as parental age, education level household income and the number of siblings.

#### Clinical examination

Clinical examination was performed by one operator (P.P.Y.L.) with the child lying supine on a table provided by the kindergarten. The operator received training and had been examining children with VPI, dmft and ICDAS examination in an outreach setting for over 4 years. Disposable dental mirrors fastened to an intra-oral light emitting diode (MirrorLite, Kudos Crown Ltd., Hong Kong) along with a WHO CPI probe were used to record the VPI score and decayed, missing, filled, surfaces (dmfs) and teeth (dmft) scores. After cleaning and drying the teeth with gauze, the carious status of the primary second molars was noted using the ICDAS coding system. As air-drying with 3 in 1 syringe was not possible in an outreaching setting, ICDAS 0 and 1 lesions were grouped together for record to enhance detection reliability. The ball end of the CPI probe was used for tactile sense to detect enamel cavities or defects amongst the pits and fissures. A 10% random sample was re-examined to monitor intra-examiner reproducibility. The kappa values for intra-examiner reliability were 0.774, 0.964 and 0.834 for VPI, dmft and ICDAS examination, respectively (Lam et al. 2021).

#### Randomisation and treatment allocation

Treatment allocation was conducted by using random numbers generated by stratified block randomisation method in a personal computer, and the children were randomly allocated to one of the two intervention groups. The random allocation was concealed and only revealed in front of the participants just before their respective allocation.

### Interventions

Immediately after oral examination, a chair-side dental assistant prepared the materials respective to the groups allocated on the random allocation list. Both interventions were provided by one operator (P.P.Y.L.). For the sealant group, GIS was applied using the finger pressure technique outlined in the WHO manual for atraumatic restorative treatment. The primary second molars were first cleaned and dried with gauze and afterwards, GC cavity conditioner (10% polyacrylic acid) was applied with micro-brush for 10–15 s and subsequently cleaned with wet cotton pellets. The assistant then mixed a capsule of standardised liquid–powder ratio GIC (GC Fuji VII, GC Asia) with the amalgamator and the operator applied the material on the occlusal surface using a plastic instrument. A gloved finger with petroleum jelly (Vaseline) was used to rub the GIC into the pits and fissures. Subsequently, excess material was removed using a hand excavator. The operator followed a standard technique and a protocol for each subject.

For the NaFV group, the operator placed 0.25 ml of varnish (Colgate Duraphat varnish, Colgate-Palmolive UK Ltd) in the plastic dappen dish. A micro-brush was used to apply the varnish onto the second primary molars and rest of the dentition. The child was instructed not to eat or drink for at least half an hour after the application.

### Assessment of dental anxiety, cooperativeness and pain

The following four parameters were assessed at baseline and post-intervention including Frankl Behaviour Rating Scale (FBRS) (Frankl 1962) and Venham Behaviour Rating Scale (VBRS) (Venham et al. 1980) for DA and cooperativeness assessment; Wong–Baker Faces Scale (WBFS) (Wong and Baker 2001) for pain assessment and the length of time for intervention. Frankl Behaviour Rating Scale is a 4-point behavioural scale which ranges 1 (definitely negative) to 4 (definitely positive). Venham Behaviour Rating Scale is a 6-point behavioural scale ranging from 0 (total cooperation) to 5 (general protest). Besides assessing children’s behaviour and reactions, these two scales may provide an insight to the patient’s inner emotions and anxiety, which are reflected by their actions. Wong–Baker Faces Scale has long been utilised in previous studies to assess pain and discomfort in the paediatric population (Garra et al. 2013). It consists of 6 sequential faces, the number 0 happy face meaning “no pain at all” whilst the number 10 crying face meaning “most intense pain imaginable”.

One assessor (H.H.C.C.) was responsible to examine the following parameters during the study period. Before any treatment or intervention, the child was first be introduced to the WBFS. This is to ensure that the children are familiar with the spectrum of faces and their corresponding meanings. Once the child is familiar with the scale, they were asked to identify a face which denotes their current state of comfort before commencement of the treatment. Afterwards, the assessor recorded the score for both FBRS and VBRS on the examination sheet before the start of treatment.

After clinical examination and random allocation of treatment group, a plastic disposable tray of dental instruments and materials were placed next to the operator by the assistant. Subsequently, a timer was started to determine the duration of the intervention. The assessor observed the child throughout the entire process and determined the score for FBRS and VBRS to signify the child’s level of DA and cooperativeness during the procedure. After the intervention was completed, the timer was stopped, and the child was asked to choose a WBFS face to denote their current sentiment after the intervention.

### Outcome measures

The primary outcome measure is the change in children’s DA and cooperativeness (FBRS and VBRS) as well as pain (WBFS) rating from the start to the end of the intervention. Secondary outcome measure is the duration of the two interventions.

### Data processing and analysis

Data analysis was be performed using SPSS® Statistics version 23.0 (SPSS Inc, Chicago, IL, USA). Data proofreading was performed after data entry to identify and correct any anomalies before analysis. Chi-square test was used to assess the difference between the two groups according to the distribution of children’s demographic characteristics, such as gender, place of birth, oral health-related behaviour, use of fluoride toothpaste, snacking habit, parent’s education and family income. A multi-level logistic regression analysis was performed to analyse the effects of independent variables on the DA level of children, measured with FBRS, VBRS and WBFS. The variables include the child’s socio-demographic characteristics, oral health-related behaviours, clinical characteristics (VPI score, dmft score) and the treatment groups. The level of statistical significance for all tests was set at 5%.

## Results

**Fig. 1:**
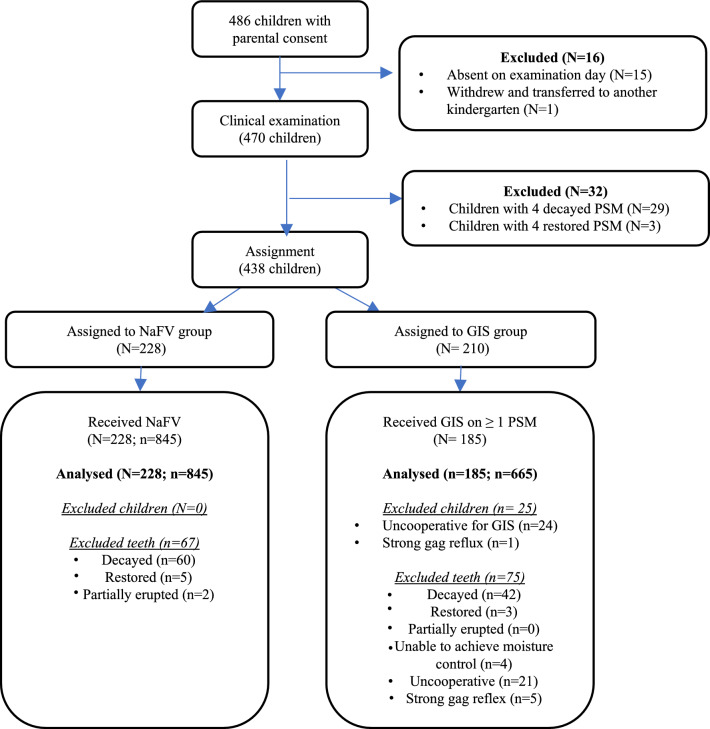
Flow of participants

A total of 486 parental consent and completed questionnaires were received, but only 470 children were being examined as 16 students were absent on the day of examination and withdrew from the study at baseline. Thirty-two children were further excluded from the study because of the following reasons: scoring ICDAS code 4 for all second primary molars (29), and all primary second molars were restored (3). A total of 438 children were randomised into the two intervention groups, with all 228 children in the NaFV group received the treatment. There were 25 children in the GIS group being further excluded due to uncooperativeness (24) and strong gag reflux (1) precluding sealant placement. Hence, 413 children (NaFV group = 228 children, GIS = 185 children) were included in the study (Fig. 1).

### Demographic background of children

No statistically significant difference was found between the children in the 2 study groups with respect to their demographic background, oral health behaviours, and oral health parameters (p > 0.05) (Table 1). However, only father’s education level showed a statistically significant difference (*p* = 0.023), with significantly more fathers from NaFV group (59.6%) attending only secondary school compared to GIS group (45.4%).

**Table 1:** Baseline demographic profile, oral health status and cooperativeness of study participants

	NaFV group *n* = 228	GIS group *n* = 185	
	% (*n*)	% (*n*)	*p* value
Child demographic profile			
Child gender			
Male	53.5 (122)	48.1 (89)	0.275^†^
Female	46.5 (106)	51.9 (96)	
Place of birth			
Hong Kong	95.2 (217)	90.8 (168)	0.079^†^
Others or undisclosed	4.8 (11)	9.2 (17)	
Grade at baseline			
Kindergarten grade 1	37.3 (85)	41.0 (59)	0.253^†^
Kindergarten grade 2	62.7 (143)	68.1 (126)	
Parent age range			
20–29 years	14.5 (33)	17.8 (33)	0.478^‡^
30–39 years	60.1 (137)	59.5 (110)	
40 years or above	22.4 (51)	21.6 (40)	
Undisclosed	3.1 (7)	1.1 (2)	
Family income (HKD per month)			
< $10.000	6.6 (15)	8.1 (15)	0.941^†^
$10,000–$20,000	25.9 (59)	22.2 (41)	
$20,001–$30,000	24.1 (55)	25.4 (47)	
$30,001–$$40,000	16.2 (37)	16.2 (30)	
$40,000 or above	19.3 (44)	21.1 (39)	
Undisclosed	7.9 (18)	7.0 (13)	
Father education level			
Primary school or less	1.3 (3)	1.1 (2)	0.111^†^
Secondary school	58.8 (134)	46.5 (86)	
Post-secondary/University	28.1 (64)	35.1 (65)	
Post-graduate or above	6.6 (15)	7.6 (14)	
Undisclosed	5.3 (12)	9.7 (18)	
Mother’s education level			
Primary school or less	3.5 (8)	1.6 (3)	0.370^†^
Secondary school	56.6 (129)	53.5 (99)	
Post-secondary/University or above	32.9 (75)	36.2 (67)	
Post-graduate or above	4.8 (11)	3.8 (7)	
Undisclosed	2.2 (5)	4.9 (9)	
Number of siblings			
Only child	25.4 (58)	35.1 (65)	0.134^†^
1 sibling	57.9 (132)	48.6 (90)	
< 1 sibling	13.2 (30)	11.4 (21)	
Undisclosed	3.5 (8)	4.9 (9)	

	Mean (SD)	Mean SD	*p* value
Age (months)	51.4 (8.2)	51.3 (8.6)	0.882^§^

Oral habits			
	% (*n*)	% (*n*)	*p* value^†^
Brushing frequency			
Less than once a day	5.7 (13)	4.9 (9)	0.309^‡^
Once a day	31.6 (72)	24.3 (45)	
Twice a day	59.6 (136)	68.6 (127)	
More than twice a day	2.2 (5)	2.2 (4)	
Undisclosed	0.9 (2)	0.0 (0)	
Supervised toothbrushing			
Never	7.0 (16)	9.2 (17)	0.581^‡^
Once a day	35.5 (81)	36.8 (68)	
Twice or more a day	56.6 (129)	53.0 (98)	
Undisclosed	0.9 (2)	1.1 (2)	
Toothpaste			
No toothpaste	7.0 (16)	4.9 (9)	0.635^‡^
Child non-fluoridated toothpaste	40.4 (92)	41.6 (77)	
Child fluoridated toothpaste	39.5 (90)	38.4 (71)	
Adult fluoridated toothpaste	0.9 (2)	2.7 (5)	
Uncertain or undisclosed	12.0 (28)	12.7 (23)	
Snacking frequency			
Less than once a day	11.4 (26)	8.1 (15)	0.359^†^
Once a day	31.6 (72)	28.6 (53)	
Twice a day	44.3 (101)	45.4 (84)	
Over twice	12.7 (29)	17.8 (33)	
Night bottle habit			
Never	65.8 (150)	68.6 (127)	0.807^†^
Yes	4.8 (11)	3.2 (6)	
Previously yes but winded up recently	6.6 (15)	5.4 (10)	
Uncertain or undisclosed	22.8 (52)	22.7 (42)	
Previous dental visit			
No	83.8 (191)	82.2 (152)	0.725^‡^
Yes	15.8 (36)	16.8 (31)	
Undisclosed	0.4 (1)	1.1 (2)	

Oral health parameters					
	Mean	Median (SD)	Mean (SD)	Median (SD)	*p* value
VPI score (%)	41.6	41.7 (16.2)	40.5	41.7(16.7)	0.520^*§*^
dmft score	1.5	0.0 (2.6)	1.4	0.0 (2.8)	0.637^¶^
dt score	1.5	0.0 (2.6)	1.4	0.0 (2.6)	0.697^¶^
mt score	0.0	0..0 (0.1)	0.0	0.0 (0.1)	0.693^¶^
ft score	0.0	0.0 (0.2)	0.0	0.0 (0.1)	0.757^¶^
dmfs score	2.1	0.0 (4.1)	2.2	0.0 (5.2)	0.767^¶^
ds score	2.1	0.0 (4.0)	2.1	0.0 (4.9)	0.776^¶^
ms score	0.0	0.0 (0.5)	0.1	0.0 (0.7)	0.693^¶^
fs score	0.1	0.0 (0.5)	0.1	0.0 (1.0)	0.511^¶^

^†^*p*-value derived from Chi-square statistics

^‡^*p*-value derived from Fisher’s exact statistics

§*p*-value derived from *t*-test for independent samples

^¶^*p*-value derived from Mann–Whitney *U* tests

*p < 0.05

**p < 0.01

***p < 0.00

### Baseline dental anxiety, cooperativeness and pain levels

No statistically significant differences in terms of baseline DA, cooperativeness and pain were found between the children amongst the two treatment groups (p > 0.05) (Table 1). 94.3% (215 children) and 95.7% (177 children) in the NaFV group and GIS group evaluated using the FBRS were deemed cooperative (*p* = 0.526). Similarly, no statistically significant differences in DA as assessed using VBRS were noted between NaFV group and GIS group as 89% (203 children) and 89.7% (166 children) scored 0, respectively (*p* = 0.820). Investigating pre-treatment pain level, WBFS also showed no significant differences between the two groups as 71.5% (163 children) and 69.2% (128 children) of the NaFV group and GIS group scored positively (score 0) (*p* = 0.945). Ultimately, baseline DA, cooperativeness and pain levels were comparatively low before commencement of intervention.

### Post-intervention dental anxiety, cooperativeness and pain

Post-intervention DA and cooperativeness measured using FBRS and pain shown through WBFS demonstrated no statistically significant differences between the two groups (*p* > 0.05). Majority of the subjects were cooperative assessed using FBRS for both NaFV and GIS groups, at 95.6% and 94.1%, respectively (*p* = 0.473). Furthermore, 57.5% and 59.5% of children did not feel the pain when rated with WBFS for both NaFV and GIS groups, respectively (*p* = 0.288). Yet, significantly more children were positively scored after NaFV application at 86.8% compared to GIS application at 77.8% using VBRS (*p* = 0.016).

### Change in dental anxiety, cooperativeness and pain levels

This trend is further displayed when analysing the change in DA, cooperativeness and pain levels (Table 2). A larger proportion of children in the GIS group became more anxious and less cooperative using VBRS at 14.1% compared to the NaFV group at 5.3% (*p* = 0.007). Such a change was not statistically significant when assessing using FBRS (*p* = 0.229), with only 3.5% and 1.6% of children had negative changes in FBRS rating in NaFV and GIS groups, respectively. Change in pain was also not significant between the two groups, with 13.6% in NaFV group and 14.6% in GIS group exhibiting deteriorated WBFS score after the intervention (*p* = 0.81). It is suggested that DA and patient behaviour assessed using VBRS displayed statistically significant differences between the two treatment groups, with children in NaFV group being more cooperative and less anxious after the treatment.

**Table 2:** Anxiety and pain levels of study participants before and after preventive interventions

	NaFV group *n* = 228	GIS group *n* = 185	
	% (*n*)	% (*n*)	*p* value
**Pre-intervention anxiety and pain**			
*Frankl scale (FBRS)*			
Cooperative (3–4)	94.3 (215)	95.7 (177)	0.526^†^
Uncooperative (1–2)	5.7 (13)	4.3 (8)	
*Venham scale (VBRS)*			
Cooperative (0)	89.0 (203)	89.7 (166)	0.820^†^
Uncooperative (≥ 1)	11.0 (25)	10.3 (19)	
*Wong–Baker scale (WBFS)*			
No hurt (0)	71.5 (163)	69.2 (128)	0.945^†^
Hurts little bit (2)	14.0 (32)	16.8 (31)	
Hurts little to even more (4–6)	6.6 (15)	6.5 (12)	
Hurts whole lot to worst (8–10)	3.9 (9)	3.2 (6)	
Cannot tell	3.9 (9)	4.3 (8)	
**Post-intervention anxiety and pain**			
*Frankl scale (FBRS)*			
Cooperative (3–4)	95.6 (218)	94.1 (174)	0.473^†^
Uncooperative (1–2)	4.4 (10)	5.9 (11)	
*Venham scale (VBRS)*			
Cooperative (0)	86.8 (198)	77.8 (144)	0.016*^†^
Uncooperative (≥ 1)	13.2 (30)	22.2 (41)	
*Wong–Baker scale (WBFS)*			
No hurt (0)	57.5 (131)	59.5 (110)	0.834^†^
Hurts little bit (2)	29.8 (68)	24.9 (46)	
Hurts little to even more (4–6)	7.9 (18)	7.6 (14)	
Hurts whole lot to worst (8–10)	0.9 (2)	3.8 (7)	
Cannot tell	3.9 (9)	4.3 (8)	
**Change in anxiety and pain**			
*Frankl scale (FBRS)*			
Negative to positive	1.8 (4)	3.8 (7)	0.229^†^
No change	94.7 (216)	94.6 (175)	
Positive to negative	3.5 (8)	1.6 (3)	
*Venham scale (VBRS)*			
Became more cooperative	3.5 (8)	2.2 (4)	0.007*^†^
No change	91.2 (208)	83.8 (155)	
Became less cooperative	5.3 (12)	14.1 (26)	
*Wong–Baker scale (WBFS)*			
Hurt more	13.6 (31)	14.6 (27)	0.810^†^
No change	58.8 (134)	60.5 (112)	
Hurt less	27.6 (63)	24.9 (46)	

Treatment time and number of included teeth			
	Mean (SD)	Mean (SD)	*p* value
Overall treatment time (min)	0.9 (2.3)	4.0 (1.3)	< 0.001***^,*§*^
Mean treatment time (per tooth)	0.2 (0.8)	1.1 (0.5)	< 0.001***^,*§*^
Mean number of teeth treated	3.9 (0.5)	3.7 (0.8)	0.006*^,*§*^

Number of teeth received intervention	% (*n*)	% (*n*)	
1	1.8 (4)	3.8 (7)	0.019*
2	2.2 (5)	7.6 (14)	
3	0.9 (2)	2.2 (4)	
4	95.2 (217)	86.5 (160)	

^*†*^*p-*value derived from Chi-square statistics

^‡^*p*-value derived from Fisher-exact statistics

§*p*-value derived from *t*-test for independent samples

*p < 0.05

**p < 0.01

***p < 0.001

### Treatment time

The overall mean treatment time for NaFV was 0.9 min (SD = 2.3) and 4.0 min (SD = 1.3) for GIS (Table 2). Furthermore, the mean treatment time per tooth was 0.2 min (SD = 0.8) and 1.1 min (SD = 0.5) for NaFV and GIS, respectively. The findings for both these parameters were found to be statistically significant (*p* < 0.001). Furthermore, there was a significant difference in number of teeth treated in the two groups (*p* = 0.019). A larger proportion of children (95.2%) in the NaFV group received the interventions for all 4 s molars whilst only 86.5% of children in GIS group completed treatment for all 4 teeth.

### Factors associated with DA and uncooperative behaviour after intervention

Multi-logistic regression was conducted to assess the effect of various demographic, social and oral health-related factors on change in cooperation after the intervention (Table 3). After regression analysis, it was found that only the interventions itself have a statistically significant effect on their child’s behaviour, with children receiving GIS more likely to displayed negative change in their behaviours (*p* = 0.032). Other socio-demographic findings and clinical findings played no significant role in the change in behaviour amongst pre-school children (p > 0.05).

**Table 3:** Factors associated with uncooperative behavior after preventive interventions

	Estimate	S.E	Odds Ratio	95% CI	*p* value
Intervention					
F-	− 1.11	0.38	0.33	(0.16, 0.69)	0.032
GIS^a^					
(Intercept)	− 1.81	0.22	0.16		< 0.001

## Discussion

Dental anxiety and other DBMP are elements a clinician must tackle when providing treatment to the paediatric population. Although DA was found to be low at 4% in a previous Hong Kong cross-sectional study (Yon et al. 2020a), other patient-reported outcomes and DBMP may influence the patient’s cooperation and hence successful delivery of preventive treatment. Similarly, both baseline and post-intervention anxiety scored with FBRS were low, ranging from 4.4 to 5.9% of the study group. This could be attributed to the outreach nature of this study. The participants were examined and operated on in a classroom setting, in the company of their peers and teachers. Such familiarity and non-clinical environment may have provided a sense of comfort for some children, and researchers should be aware that treatment delivered under outreach circumstances may evoke less anxiety compared to a dental clinic setting.

In terms of DA and cooperativeness measured with FBRS and pain measured with WBFS, no statistically significant difference was observed at baseline and post-intervention when comparing NaFV and GIS groups (Lam et al. 2024). Moreover, no significant difference was noted in scoring change of these two scales when comparing the two treatment groups. This finding could be due to the minimally invasive nature of these two treatment approaches. According to the FDI policy statement on MID, fissure sealants and fluoride varnishes are hallmarks of MID outlined by the organisation (FDI World Dental Federation 2017). Both treatment modalities do not require tooth preparation using rotary handpieces, local anaesthesia application or rubber dam application, which may induce a significant amount of distress and discomfort for patients. Thus, it is logical that NaFV and GIS application may provoke similarly low levels of anxiety and discomfort in paediatric patients.

This clinical study requires an appropriate method of assessing DA, cooperativeness and pain as these measures may influence the interpretation of results. Frankl Behaviour Rating Scale and VBRS are observational behavioural scales utilised to assess DA and cooperativeness of the children (Yon et al. 2020b). Compared to other forms of assessment such as physiological-based, the above scales do not require extra monitoring devices such as heart rate monitor, which may provoke further anxiety and uneasiness. Moreover, such observation-based scales could measure the behavioural component of anxiety whilst being more objective as specific criteria could be outlined (Yon et al. 2020b). The scales that assess DA, based on observations of clinical behaviour, are particularly advantageous when assessing young pre-school children who are not able to self-report their own fear levels (Wong 2017).

Frankl Behaviour Rating Scale is also a widely used and recognised anxiety assessment scale, and a high correlation and an association have been previously reported between FBRS and other anxiety assessment measures (Tiwari et al. 2021). Literature has demonstrated high validity and correlation between FBRS and VBRS when assessing paediatric behaviour during dental treatment (Cademartori et al. 2017). Interestingly, the current study indicates a disparity in the interpretation of anxiety between these two scales, with VBRS showing more anxiety in the GIS group. This finding could be due to the inherent descriptive nature of VBRS. FBRS is a four-point scale whilst VBRS is a six-point scale. VRBS includes five categories (scores 1–5) of increasingly uncooperative and anxious behaviour, ranging from “mild verbal protest and crying” in lower scores to “disruptive protest” that needs “physical restraint” in higher scores. On the other hand, score 0 denotes complete cooperation. With such stringent descriptions, it is easier to identify anxious and uncooperative behaviour, which may result in a significant finding with regard to VBRS. Fundamentally, the definition of cooperative behaviour may vary between scales. If one considers cooperation as total obedience as outlined by VBRS, the results illustrate that NaFV causes less anxiety and uncooperative behaviour compared to GIS. As there were more children showing no change in behaviour or become less anxious following fluoride varnish application, hence, the first null hypothesis that there is no difference in change of DA level between placement of GIS and topical application of NaFV in pre-school children has to be rejected.

Wong–Baker Facial Scale includes multiple facial expression drawings and current literature suggests that faces scales are the preferred method of pain reporting by children (Keck et al. 1996). The validity of WBFS has also been demonstrated and a high correlation to other pain scales has been reported as well (Garra et al. 2010). The effectiveness of WBFS depends on the child’s ability to understand the connotations and meaning behind the faces. Researchers attempted to explain the scale, yet some young participants still could not choose a face. One should recognise the inherent limitations of utilising self-report assessment scales. Developmental maturity and levels of comprehension may differ between pre-school students of different grades. Hence, reliability of self-reporting should be prudently assessed. Results indicated no significant differences in change of pain levels between the two treatment groups (*p* = 0.945) with 57.5% and 59.5% of subjects selecting “No hurt” after the NaFV and GIS application, respectively. Hence, the second null hypothesis that there is no difference in change of pain levels between placement of GIS and NaFV application could not be rejected.

Treatment time was found to be significant as GIS application took up significantly more time compared to NaFV application. Hence, the third null hypothesis that there is no difference in time of procedure between NaFV and GIS has to be rejected. This finding has been reported previously in studies comparing treatment time of the two preventive measures. Splieth et al. (2001) found that fluoride varnish application only took a third of the time that of fissure sealant application (Splieth et al. 2001). It is worthy to note that the disparity might be further amplified due to the outreach nature of the investigation as the patient positioning and instrument handling are affected. In a clinical setting, the time of GIS application may be shorter as conditions are more optimal to provide dental treatment. Younger children typically have shorter attention spans that lead to deteriorating behaviours over the course of treatment (Aminabadi et al. 2009). Implementing preventive treatments with shorter durations can be advantageous in optimising children’s behaviours and creating a more pleasant treatment experience.

Multi-logistic regression identified socio-demographic factors and clinical findings had no significant influence on the change in pre-school children’s behaviours when receiving preventive treatments. Such findings coincided with another study conducted in Hong Kong (Yon et al. 2020a), which also did not find any significant links between these factors except age. Varying ages of the subjects could have affected the data as the ability to effectively communicate feelings, thoughts and pain may differ between a 3-year-old and a 5-year-old. However, age was not found to be a significant factor in this study as a majority of children were recruited at similar age during in late kindergarten grade 1 to early kindergarten grade 2. Further investigations of the influence of age towards acceptance of different preventive dental treatments can be carried out when performing similar studies amongst children of different grades or when the cohort is being followed up.

Past medical or dental experiences are known factors that can influence dental anxiety in children (Campbell et al. 2011; Peng et al. 2024). However, since the participants recruited for this study were generally healthy and had no prior dental experiences, these factors were not found to be significant in the current study. Previous research has indicated that children from single-child households or with lower household incomes may exhibit higher levels of dental anxiety (Peng et al. 2024). Still, these variables were not found to be significant in the present study. Whilst parental dental anxiety levels and parenting styles have been suggested to be linked with dental anxiety in children, they were not investigated in this study due to conflicting results in the existing literature and variations in how these factors are measured and assessed (Peng et al. 2024; Lee et al. 2018), as well as their potentially correlations with other confounding factors, such as past dental experience, age and socio-economic status (Peng et al. 2024; Lee et al. 2018). Nonetheless, future investigations on dental behaviours related to preventive treatments could consider examining the impact of these factors.

Certain limitations exist for the current investigation and should be discussed. Firstly, there were 25 students assigned to the GIS group being inevitably excluded as GIS could not be successfully placed on their molars. The reasons behind the failure of GIS placement were due to uncooperative behaviours (*n* = 24) and strong gag reflex (*n* = 1). Meanwhile, no children assigned to the NaFV have been excluded due to uncooperative behaviours as NaFV required minimal cooperation from the children during the short application process. Potential selection bias may have been introduced as these non-compliant children initially assigned to the GIS group had to be excluded from the analysis. This exclusion could have resulted in an underestimation of dental fear results for the GIS group, potentially introducing bias to the overall study findings. Whilst both VBRS and FBRS are well-validated assessment tools that have been extensively utilised in clinical trials (Yon et al. 2020b), they remain indirect methods for evaluating DA in children as they primarily focus on behaviours rather than anxiety levels. In the present study, VBRS and FBRS were administered by trained and calibrated researchers who assisted in the intervention applications. Despite these measures, there is still a risk of outcome assessment bias in postoperative behaviours due to challenges in achieving blinding. These limitations may compromise the reliability and the validity of the dental fear assessments employed. Furthermore, the current study only utilised self-report assessments and observational assessments. Physiological assessments, considered the most objective measure of anxiety (Yon et al. 2020b), were not employed due to the challenges associated with monitoring equipment in an outreach setting. A more comprehensive and balanced approach to assessing dental anxiety would have been advantageous, incorporating physiological assessments alongside self-reported and observational assessments.

Clinicians may recognise the higher levels of anxiety, pre-cooperative behaviour and longer treatment time associated with fissure sealant placement. The decision to apply fluoride varnish, fissure sealant or both should depend on caries risk, tooth anatomy and individual patient cooperation. Ultimately, both treatment modalities are important aspects of preventive care in managing pre-cooperative children with dental caries.

## Conclusion

Considering the limitations of the present study, the following conclusions can be made:

Pre-school children exhibited higher levels of dental anxiety and uncooperative behaviour during fissure sealant application compared to fluoride varnish application even though similar pain levels were reported. The application time for glass ionomer fissure sealants on primary second molars was found to be four times longer than that for sodium fluoride varnish. Although quarterly-applied fluoride varnish and single application of glass ionomer fissure sealants demonstrate comparable preventive efficacy in preventing occlusal caries amongst primary second molars, quarterly-applied fluoride varnish may be more suitable for young children exhibiting elevated levels of dental anxiety and uncooperative behaviour.

## Data Availability

The data that support the findings of this study are available from the corresponding author upon reasonable request.
